# Pediatric anesthesia during the coronavirus disease epidemic – One pediatric surgical hospital's rapid transition back to care

**DOI:** 10.1111/pan.14000

**Published:** 2020-11-03

**Authors:** Sampaguita Tafoya, Sundeep Tumber

**Affiliations:** ^1^ Department of Anesthesia Shriners Hospitals for Children Northern California Sacramento California USA

Shriners Hospitals for Children ‐ Northern California provides highly specialized pediatric surgical care. The rapid spread of coronavirus disease 2019 (COVID‐19) required timely suspension of our elective surgeries. Mitigating disease spread and protecting patients and staff became paramount. Because pediatric patients in particular carry and may spread SARS‐CoV‐2 while asymptomatic, and due to the large amount of respiratory a

erosols produced during airway manipulation, an unprecedented risk of provider infection came to exist. Surgical cases were tiered based on urgency and only emergent or urgent cases proceeded using airborne precaution personal protective equipment (PPE).^1^ Furthermore, asymptomatic patients infected with SARS‐CoV‐2 undergoing surgery have higher postoperative morbidity and mortality.^2^ However, surgery in children is rarely completely elective and could not be postponed indefinitely. The ability to resume pediatric surgical care during this pandemic would only be possible if a completely new path was forged.

Once local COVID‐19‐infected cases did not peak as projected, it became clear that the road forward would require a multidisciplinary onsite preoperative testing program. A core nursing group was trained for specimen collection to ensure reliable sampling. Access to test equipment from our local academic institution was vital. In‐house validation demonstrated both the sensitivity and specificity of their Roche Diagnostics cobas^®^ SARS‐CoV‐2 PCR Test to be >99%. Ultimately, two anesthesiologists were responsible 24‐7 for test ordering and case management.

Reopening requires a daily assessment of one's local COVID‐19‐infected case prevalence. We based our ability to proceed on the nearby academic institution's coronavirus ICU census, to provide a surrogate indication of the pandemic status in our community and whether or not our specialty surgical hospital could appropriately ramp up operations.

An assessment of available PPE continues to be a critical factor in continuing operations. Initially, N95 masks were extremely limited. We utilized reusable powered air‐purifying respirators (PAPRs) for the majority of cases. Though very time consuming, this allowed us to conserve our more critical supply of N95s. As PPE supply stabilized, our ability to increase surgical volume improved.

All add‐on cases were presented to the anesthesia board runner. Prior to the pandemic, many would proceed to the operating room within 24 hours. Once COVID‐19 testing began, if no imminent morbidity was expected, surgery was delayed 24‐48 hours to allow for testing. A previously rigid surgical block system shifted to an urgency‐based schedule to allow add‐ons of this nature to be accommodated.

Recommendations were recently provided by the American Society of Anesthesiologists regarding perioperative testing and PPE.^3^ We developed a COVID‐19 Perioperative Testing Decision Tree (Figure 1) to guide appropriateness for surgery and PPE recommendations. Due to our low regional coronavirus disease incidence, our access to highly sensitive and specific testing, and a reliable sample collection processes, we opted to use droplet precaution PPE (face mask and eye protection)^1^ during aerosol generating procedures in children who tested negative and were asymptomatic within 48 hours preoperatively. This allowed for conservation of scarce airborne precaution PPE (N95 or higher level respirator).^1^

**Figure 1:**
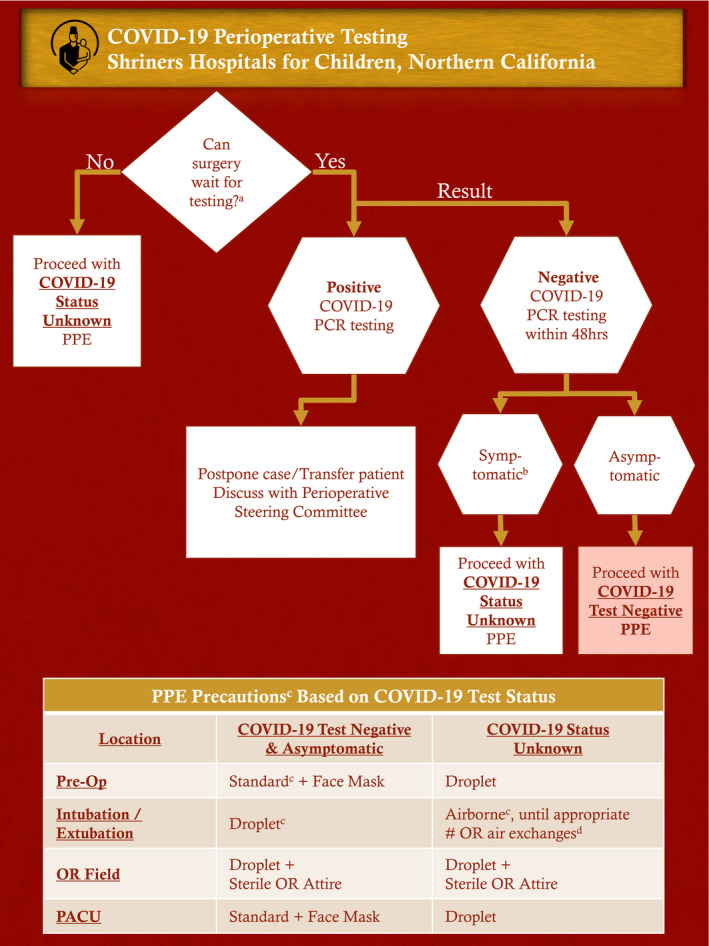
COVID‐19 Perioperative Testing Guiding Decision Making and PPE. Abbreviations: PPE, personal protective equipment; Pre‐Op, preoperative holding area; OR, operating room; PACU, post‐anesthesia care unit. ^a^If disagreement, discussion with Chief of Anesthesia, Chief of Surgical Service, and potentially, Perioperative Steering Committee. ^b^Defined as fever >100.4°C or cough. ^c^Centers for Disease Control and Prevention, Transmission‐Based Precautions.^1^ ^d^Centers for Disease Control and Prevention, Infection Control, Appendix B Air^5^

Since reopening in mid‐April, we have tested over 350 children. Two asymptomatic patients tested positive necessitating reevaluation of surgical urgency. An acute burn patient was treated with wound care instead of immediate excision and grafting. After 10 days and two negative tests were completed,^4^ that patient came to the operating room. One fracture case was treated with an alternate plan of casting in a negative pressure room with minimal sedation. A small subset of cases were too urgent to await testing and airborne PPE was utilized. Overall, we have proceeded with the majority of cases without the use of airborne precaution PPE, thus conserving resources and minimizing transmission risk.

Unanticipated hurdles arose. Surgical tiering based on urgency preferentially placed medically complex patients on the schedule in the initial weeks of reopening. Preoperative optimization resources needed to be rapidly coordinated in a setting of pediatric specialists that were not performing routine visits. Blood shortages resulted in postponement of select cases, such as spine fusion for neuromuscular scoliosis.

We continue to adapt to the changing landscape of this pandemic. Our supply of testing reagent remains uncertain. Rapid, accurate in‐house testing is an immediate goal. Overall, this timely reopening response took considerable physician leadership. With such, we have been able to resume providing optimal care while protecting patients and providers, conserving PPE, and lessening the potential for morbidity associated with receiving an anesthetic during the COVID‐19 pandemic.

## CONFLICT OF INTEREST

The authors report no conflict of interest.
